# Identification of a Circadian Clock-Controlled Neural Pathway in the Rabbit Retina

**DOI:** 10.1371/journal.pone.0011020

**Published:** 2010-06-10

**Authors:** Christophe Ribelayga, Stuart C. Mangel

**Affiliations:** Department of Neuroscience, The Ohio State University College of Medicine, Columbus, Ohio, United States of America; Tufts University, United States of America

## Abstract

**Background:**

Although the circadian clock in the mammalian retina regulates many physiological processes in the retina, it is not known whether and how the clock controls the neuronal pathways involved in visual processing.

**Methodology/Principal Findings:**

By recording the light responses of rabbit axonless (A-type) horizontal cells under dark-adapted conditions in both the day and night, we found that rod input to these cells was substantially increased at night under control conditions and following selective blockade of dopamine D_2_, but not D_1_, receptors during the day, so that the horizontal cells responded to very dim light at night but not in the day. Using neurobiotin tracer labeling, we also found that the extent of tracer coupling between rabbit rods and cones was more extensive during the night, compared to the day, and more extensive in the day following D_2_ receptor blockade. Because A-type horizontal cells make synaptic contact exclusively with cones, these observations indicate that the circadian clock in the mammalian retina substantially increases rod input to A-type horizontal cells at night by enhancing rod-cone coupling. Moreover, the clock-induced increase in D_2_ receptor activation during the day decreases rod-cone coupling so that rod input to A-type horizontal cells is minimal.

**Conclusions/Significance:**

Considered together, these results identify the rod-cone gap junction as a key site in mammals through which the retinal clock, using dopamine activation of D_2_ receptors, controls signal flow in the day and night from rods into the cone system.

## Introduction

The remarkable ability of the vertebrate retina to adapt to the ∼10^9^–fold range of light intensities that spans a moonless night and a bright sunny day relies on a complex interplay between responses to the mean background illumination and signals originating from an endogenous circadian (24-h) clock [1]–[3]. Although the clock acts in synchrony with the light/dark cycle, its activity persists in constant darkness, thereby providing an endogenous reliable mechanism that anticipates the changes in background illumination that occur in the day and night.

Although the circadian clock in the mammalian retina regulates many physiological processes in the retina, including increasing dopamine release in the day [2], [4], [5], it is not known whether and how the clock controls the neuronal pathways involved in visual processing in the day and night. Recent evidence in the fish [6], [7] indicates that circadian modulation of the dopamine D_2_ receptors on rod and cone photoreceptor cells controls whether the gap junctions between rods and cones [1], [8] are functionally open or closed. By controlling the rod-cone gap-junctional conductance, so that electrical communication between rods and cones is weak during the day when dopamine levels are high and robust at night when dopamine levels are low [6], the clock modulates rod input to cones [6] and cone-connected second-order neurons [7]. Due to the clock-induced increase in the conductance of the electrical synapses between rods and cones at night, fish cones can respond to very dim light stimuli (scotopic range, see definition in Materials and Methods) because of the signals they receive from coupled rods [6] and can transmit these signals to cone-connected horizontal cells [7], a type of second-order neuron that is postsynaptic to cones, but not to rods [9], [10]. In contrast, during the day when electrical communication between fish rods and cones is minimal, cones and cone-connected horizontal cells cannot respond to dim light stimuli in the scotopic range.

In the mammalian retina, however, although it has been shown that tracer coupling between mouse rods and cones is greater at night than in the day [6], it is not known whether and how the clock controls rod pathway function (e.g. signaling from rods to second-order neurons). Here, we show in the rabbit retina (see Materials and Methods for a discussion of our choice of species) that the light responses of axonless (A-type) horizontal cells depend on the time of day and are under the control of the retinal clock. Specifically, under dark-adapted conditions, these second-order cells respond to very dim light in the low scotopic range at night, but to mesopic light (see definition in Materials and Methods) in the day, demonstrating that rod input to A-type horizontal cells substantially increases at night. We further show that under dark-adapted conditions the extent of rod-cone neurobiotin tracer coupling in the rabbit retina is minimal during the day and maximal at night, but that rabbit A-type horizontal cells are extensively coupled to each other in both the day and night. Finally, we show that the retinal clock uses dopamine D_2_, but not D_1_, receptor activation to control rod-cone coupling and the light responses of A-type horizontal cells. These observations thus identify a clock-controlled neural pathway in the mammalian retina in which the retinal circadian clock uses dopamine to activate D_2_ receptors, thereby controlling rod-cone coupling and the flux of rod signals into the cone pathways. Due to the action of the endogenous circadian clock in the mammalian retina, at night (but not in the day), rods are able to signal dim light information to cones, which can then signal their postsynaptic targets.

## Results

### Circadian clock control of the light responses of rabbit A-type horizontal cells

**Figure 1:**
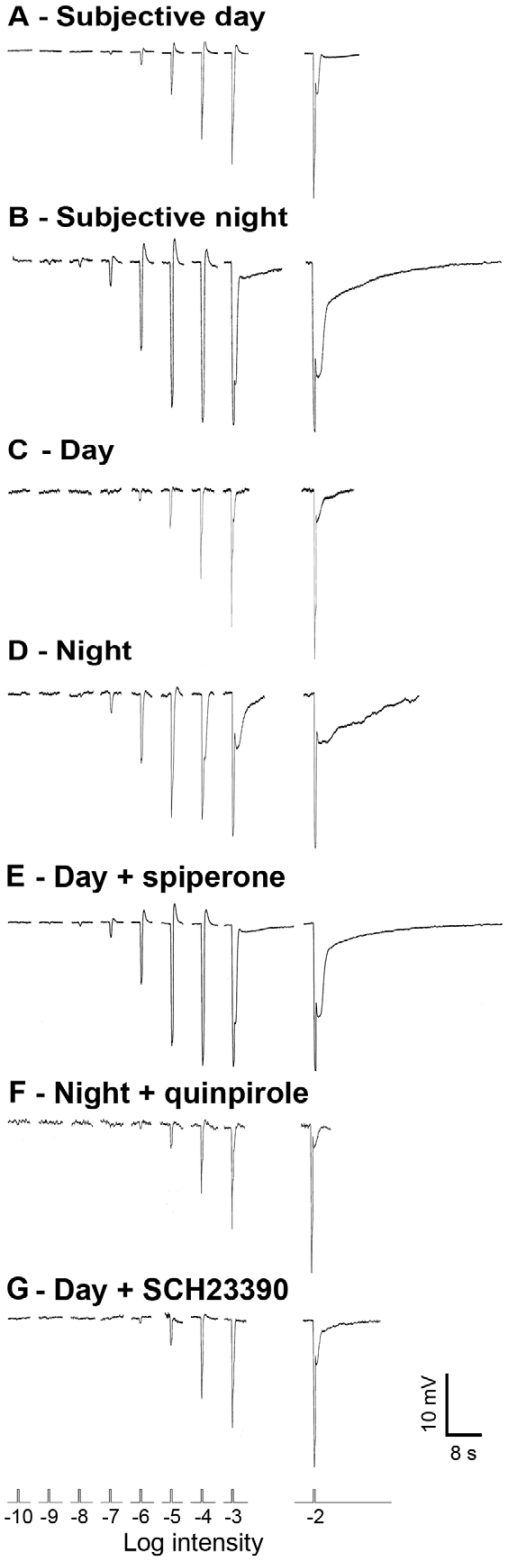
The retinal circadian clock uses dopamine and D_2_ receptors to control the light responses of rabbit A-type horizontal cells. *A-G*, Representative examples of A-type horizontal cell responses to a series of 500 ms full-field white light stimuli of increasing intensity recorded under dark-adapted conditions during the subjective day (*A*), the subjective night (*B*), the day (*C*), the night (*D*), the day in the presence of the D_2_ dopamine receptor antagonist spiperone (10 µM) (*E*), the night in the presence of D_2_ dopamine receptor agonist quinpirole (1 µM) (*F*), and the day in the presence of the D_1_ dopamine receptor antagonist SCH23390 (10 µM) (*G*). The light responses of only 1 cell per retina to the full series of light intensities were recorded.

The light responses of A-type horizontal cells in superfused rabbit retinas were studied under thoroughly dark-adapted conditions (background *I*<−11 log *I*_o_) during the subjective day (circadian time (CT)2–10) and subjective night (CT14–22) of a circadian cycle and during the day (zeitgeber time (ZT)2–10) and night (ZT14–22) of a regular light-dark cycle [see Materials and Methods]. Figure 1 shows typical examples of the light responses of dark-adapted A-type horizontal cells to full-field white light stimuli of different intensities recorded during the subjective day (Fig. 1A), subjective night (Fig. 1B), day (Fig. 1C), and night (Fig. 1D). The recorded cells were identified as A-type horizontal cells based on morphological criteria, following the injection and visualization of neurobiotin tracer. During the subjective day and day, A-type horizontal cell light responses were similar to those reported in previous studies [11]–[14]. Their light responses exhibited an initial transient peak followed by a hyperpolarizing after-potential. The amplitude of the former and the duration of the latter increased with light intensity and were particularly prominent at high photopic [see definition in Materials and Methods] intensities (*I*>−4 log *I*_o_) (Figs. 1A, C). Under our conditions and using a 0.5-mV criterion, A-type horizontal cells had a light response threshold of ∼−6.5 log *I*_o_ during the subjective day and day (Figs. 1A, C). In contrast, during the subjective night and night (Figs. 1B, D), their light response threshold was ∼−8.0 log *I*_o_.

**Figure 2 pone-0011020-g002:**
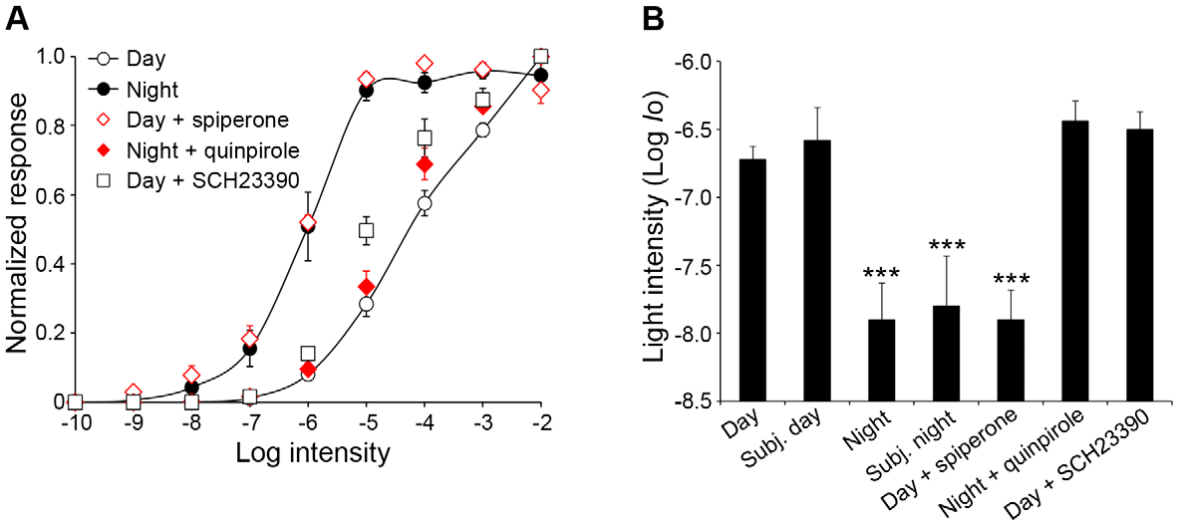
Light response amplitude and sensitivity of rabbit A-type horizontal cells vary with the time of day and D_2_ receptor activity. *A,* Average normalized intensity-response curves of A-type horizontal cells recorded under dark-adapted conditions during the day (*n* = 8) and subjective day (*n* = 6) (open circles, solid line), night (*n* = 4) and subjective night (*n* = 3) (filled circles, solid line), and in the day in the presence of spiperone (10 µM; open diamonds; *n* = 9) or SCH23390 (10 µM; open squares; *n* = 8), and the night in the presence of quinpirole (1 µM; filled diamonds; *n* = 8). Two-way ANOVA analysis revealed both intensity and condition effects for each response property measured. Data points represent averaged data from *n* cells (1 cell/retina) ± SEM. *B*, Average light response threshold (i.e. intensity required to elicit a 0.5 mV response) of A-type horizontal cells under the conditions described in (*A*). Data points represent averages of 5 to 25 measurements. ***, *P*<0.001 compared to day (Tukey's post test).

Because similar day/night differences in the light responses of dark-adapted A-type horizontal cells were observed under both circadian conditions (i.e. prolonged dark adaptation >12 h; Figs. 1A and 1B) and during a regular light/dark cycle (i.e. dark adaptation >1 h; Figs. 1C and 1D), as illustrated for light response thresholds (see Fig. 2B), the data were pooled into 2 groups: day-dark-adapted (i.e. day and subjective day data) and night-dark-adapted (night and subjective night data). Figure 2 and Table 1 compare the average light response properties of A-type horizontal cells in the day and night using these two groups. The averaged data reveal that the intensity to generate a half-maximal amplitude response was significantly greater during the day than at night (Fig. 2A, Table 1). In addition, the average light response threshold of the cells was ∼1.5 log unit lower at night than during the day (Fig. 2B, Table 1). The higher sensitivity at night in the low scotopic range indicates that rod input to horizontal cells substantially increases at night, compared to the day.

**Table 1:** Light response properties of dark-adapted rabbit A-type horizontal cells under different experimental conditions.

Response property	Day	Night	Day+spip.	Night+quin.	Day+SCH	ANOVA (*P* value)
RMP (mV±SEM)	−32.6±0.6	−31.7±0.9	−33.8±2.0	−31.5±0.9	−31.4±0.8	*F*_4,45_ = 0.779 (0.545)
HMAI (log *I*_o_±SD)	−4.05±0.90	−6.11±1.23***	−6.17±0.82***	−4.52±0.80	−5.00±0.96	*F*_4,45_ = 10.1 (<0.0001)
sample size (*n*)/fit (*r* ^2^)	14/0.96	7/0.93	9/0.96	8/0.97	8/0.97	
*λ*_max_ (nm±SD)	502±2	500±3	503±3	500±2	503±3	*F*_4,40_ = 2.27 (0.081)
peak sensitivity (*k*±SD)	−8.45±0.06	−7.38±0.10***	−7.49±0.08***	−8.50±0.06	−8.44±0.07	*F*_4,40_ = 464 (<0.0001)
MIR (Rh*.rod^−1^.s^−1^±SD)^a^	1.51±0.01	0.13±0.002	0.17±0.002	1.69±0.01	1.48±0.01	
sample size (*n*)/fit (*r* ^2^)	19/0.93	8/0.93	6/0.95	5/0.98	3/0.98	

Experimental data are averages ± SEM and residues from non-linear analysis are averages ± SD.

***P<0.001 compared to day value (Tukey *post-hoc* test).

a the mean isomerization rate per rod (MIR) was calculated from the peak sensitivity *k* (see Materials and Methods). RMP: resting membrane potential; HMAI: half-maximal amplitude intensity.

To test whether the circadian clock uses dopamine to regulate the light responses of A-type horizontal cells in the rabbit retina, we tested the effects of spiperone, a selective antagonist of the dopamine D_2_ receptor family, on horizontal cell light responses during the day under dark-adapted conditions, when extracellular dopamine levels are high [4], [5]. Application of spiperone (10 µM; >1 h) affected the light responses of A-type horizontal cells so that they resembled those typically recorded at night, as shown by a representative example of the light responses of a single cell (Fig. 1E) and by averaged data (Fig. 2, Table 1). In contrast, application of quinpirole (1 µM; >1 h), a selective agonist of the D_2_ receptor family, at night reversed the effects of the clock and the light responses of A-type horizontal cells resembled those typically recorded during the day (Fig. 1F-light responses of a single cell; Fig. 2, Table 1-averaged data). Finally, application of the D_1_ receptor antagonist SCH23390 (10 µM; >1 h) during the day had no effect on any of the light response properties of A-type horizontal cells (Fig. 1G-light responses of a single cell; Fig. 2, Table 1-averaged data). We conclude that the clock increases dopamine levels and D_2_, but not D_1_, receptor activation in the outer retina during the day, so that rod input to A-type horizontal cells is greatly reduced.

**Figure 3 pone-0011020-g003:**
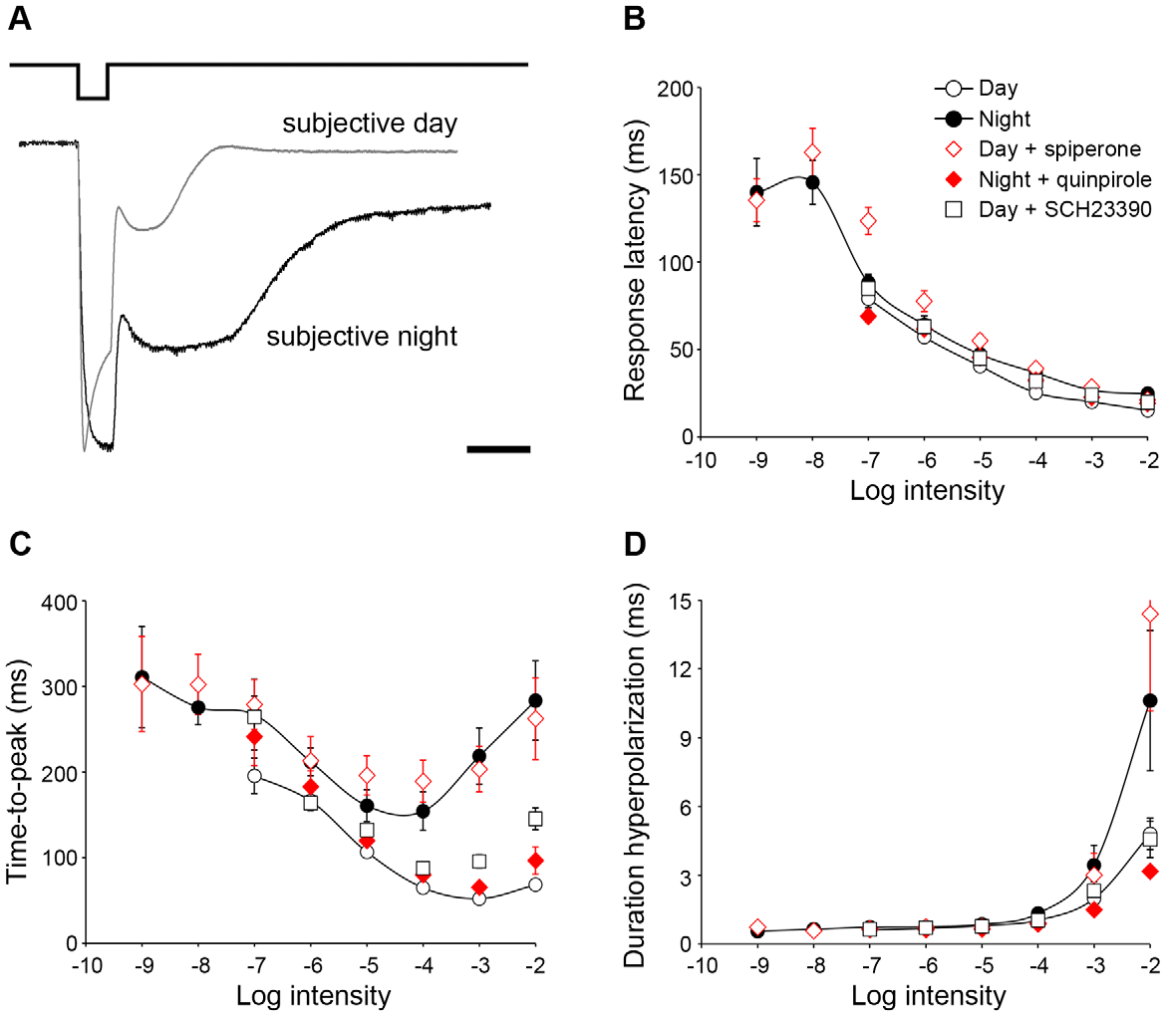
Light response kinetics of rabbit A-type horizontal cells varies with the time of day and D_2_ receptor activity. *A*, Representative examples of A-type horizontal cell responses to a light stimulus flashed (500 ms) at intensity −2 log *Io* recorded during the subjective day (gray trace) and subjective night (black trace). The amplitude of each response has been normalized relative to its peak for better comparison of the traces. Scale bar: 1 s. *B*, Average latency, *C*, time-to-peak, and *D*, total duration of the hyperpolarizing portion of A-type horizontal cell light responses recorded under dark-adapted conditions during the night and subjective night (filled circles, solid line; *n* = 7), day and subjective day (open circles, solid line; *n* = 14), and day in the presence of spiperone (open diamonds; *n* = 9) or SCH23390 (open squares; *n* = 8), and night in the presence of quinpirole (filled diamonds; *n* = 8). Two-way ANOVA analysis revealed both intensity and condition effects for each response property measured. See Materials and Methods for definitions of response measures. Data points represent averaged data from *n* cells (1 cell/retina) ± SEM.

The time course of A-type horizontal cell light responses was also different in the day and night following dark adaptation. Figure 3A illustrates typical examples of A-type horizontal cell responses to a flash of light at the same bright (photopic) intensity (−2 log *I*_o_) recorded during the day and night. Comparison of the normalized traces reveals that the time-to-peak and the duration of the responses were greater at night compared to the day, findings confirmed by the averaged time-to-peak (Fig. 3C) and response duration (Fig. 3D) data, especially at high light intensities. The slow time course of the responses at night (Figs. 1B, 1D, 3) is consistent with substantial rod input to A-type horizontal cells at night. The averaged data also show that spiperone application during the day under dark-adapted conditions altered the time course of the light responses of A-type horizontal cells so that they resembled those typically recorded at night (Figs. 3C, 3D) and that quinpirole application at night under dark-adapted conditions altered the time course of the light responses so that they resembled those typically recorded during the day (Figs. 3C, 3D). In contrast, SCH23390 application during the day under dark-adapted conditions had no effect on the time course of the light responses (Figs. 3B–D).

**Figure 4:**
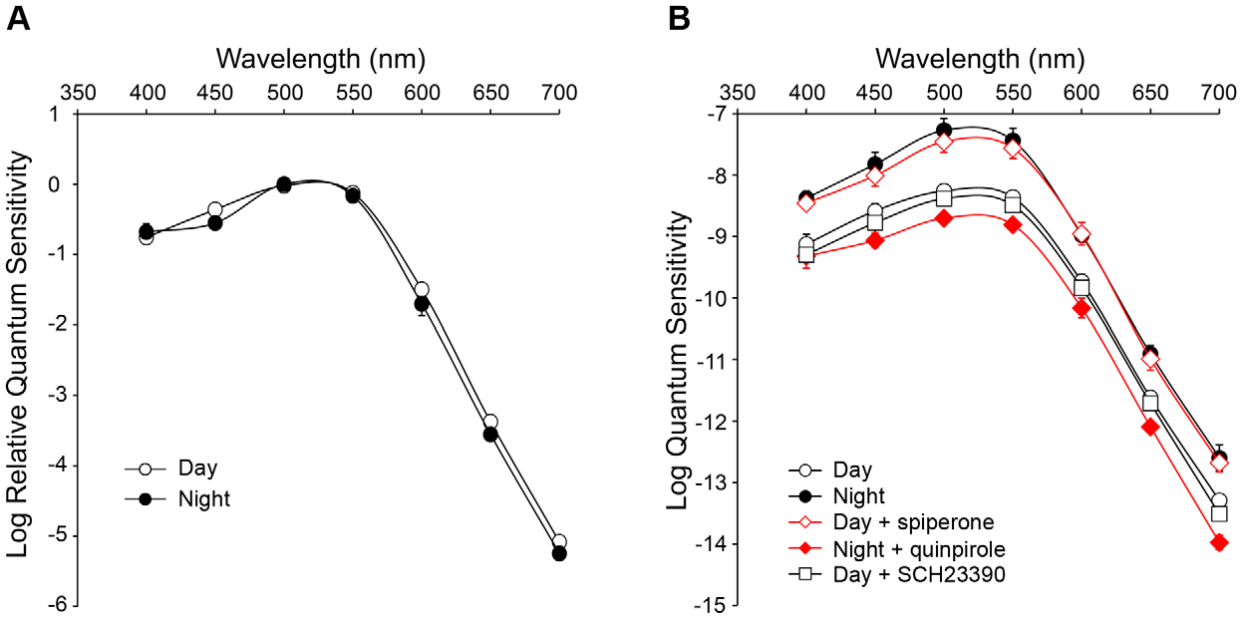
The circadian clock uses dopamine D_2_ receptors to regulate the spectral sensitivity of rabbit A-type horizontal cells. *A*, Relative spectral sensitivity of A-type horizontal cells recorded under dark-adapted conditions during the day (open circles; *n* = 19), and the night (filled circles; *n* = 8). *B*, Absolute spectral sensitivity of A-type horizontal cells recorded under dark-adapted conditions during the night (filled circles; *n* = 8), day (open circles; *n* = 19), and day in the presence of spiperone (10 µM; open diamonds; *n* = 6) or SCH23390 (10 µM; open squares, *n* = 3), and night in the presence of quinpirole (1 µM; filled diamonds; *n* = 5). Data points represent average sensitivity from *n* cells (1 cell/retina) ± SEM.

The spectral sensitivity of dark-adapted A-type horizontal cell light responses was determined in the day and night. Based on a 0.5 mV response criterion, the peak spectral sensitivity was measured as ∼500 nm (λ_max_) during both the day and night (Fig. 4A, Table 1). Although the relative contribution of rods and cones to the light responses of A-type horizontal cells cannot be established based on λ_max_, because the spectral sensitivities of rabbit rods (λ_max_ ∼500 nm) and middle-wavelength cones (λ_max_ ∼509 nm) greatly overlap [15], the quantum sensitivity (*k*) of A-type horizontal cells to green light (500 nm) was increased by ∼1 log unit at night (Fig. 4B, Table 1). Using a 0.5 mV criterion, we estimated that the response threshold of dark-adapted A-type horizontal cells at the peak sensitivity (500 nm) corresponds to a mean isomerization rate per rod of ∼1 R*.rod^−1^.s^−1^ during the day and ∼0.1 R*.rod^−1^.s^−1^ at night (Table 1, see Materials and Methods). Our data thus indicate that A-type horizontal cells respond to low scotopic light at night and mesopic light under dark-adapted conditions during the day. Considered together, these data are consistent with a clock-controlled increase in rod input to A-type horizontal cells at night.

### Circadian changes in rod-cone, but not horizontal cell-horizontal cell, tracer coupling

**Figure 5:**
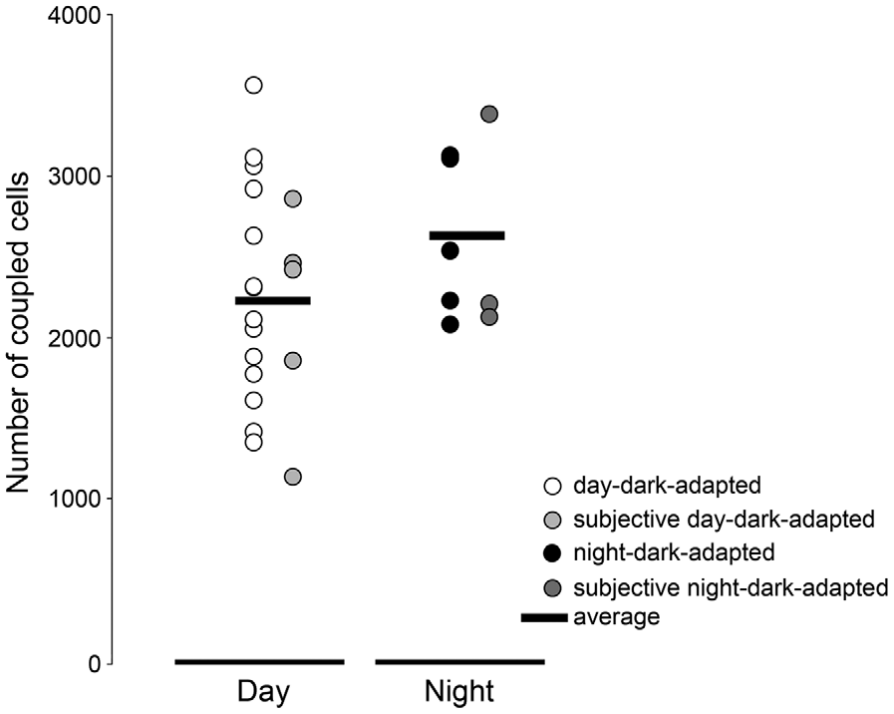
Tracer coupling between rabbit A-type horizontal cells does not vary with the time of day. Extent of A-type horizontal cell tracer coupling under dark-adapted conditions in the subjective day (*n* = 5; filled light grey circles) and day (*n* = 14; open circles) and in the subjective night (*n* = 3; filled dark grey circles) and night (*n* = 5; filled black circles). Data were pooled into 2 groups (day-dark-adapted and night-dark-adapted) and averaged (horizontal bars). No difference was found between the 2 groups (Student's t-test; *P* = 0.201). Data points represent averaged number of coupled cells from *n* cells (1 cell/retina) ± SEM. For these experiments, light stimuli were never brighter than −5 log *I*_o_.

Rabbit A-type horizontal cells are extensively coupled to each other through gap-junctions [14], [16]. Moreover, mammalian horizontal cell coupling is dynamically regulated by dopamine and changes in coupling strength shape horizontal cell receptive fields and light responses [17], [18]. To determine whether the effects of the clock on the light responses of A-type horizontal cells might result from changes in horizontal cell coupling, the extent of neurobiotin coupling was examined and found not to change under dark-adapted conditions during the day, night, subjective day and subjective night (Fig. 5). When pooled into two groups, dark-adapted-day and dark-adapted-night, tracer coupling averaged 2243±151 (SEM) (*n* = 19) and 2587±185 cells (*n* = 10), respectively. In addition, we found no difference in the resting membrane potential in the day and night (Table 1). Taken together, these observations do not provide evidence for a post-synaptic origin of the day/night variations in the light response properties of A-type horizontal cells and suggest that the clock does not act directly on the cells themselves but rather on their input.

**Figure 6:**
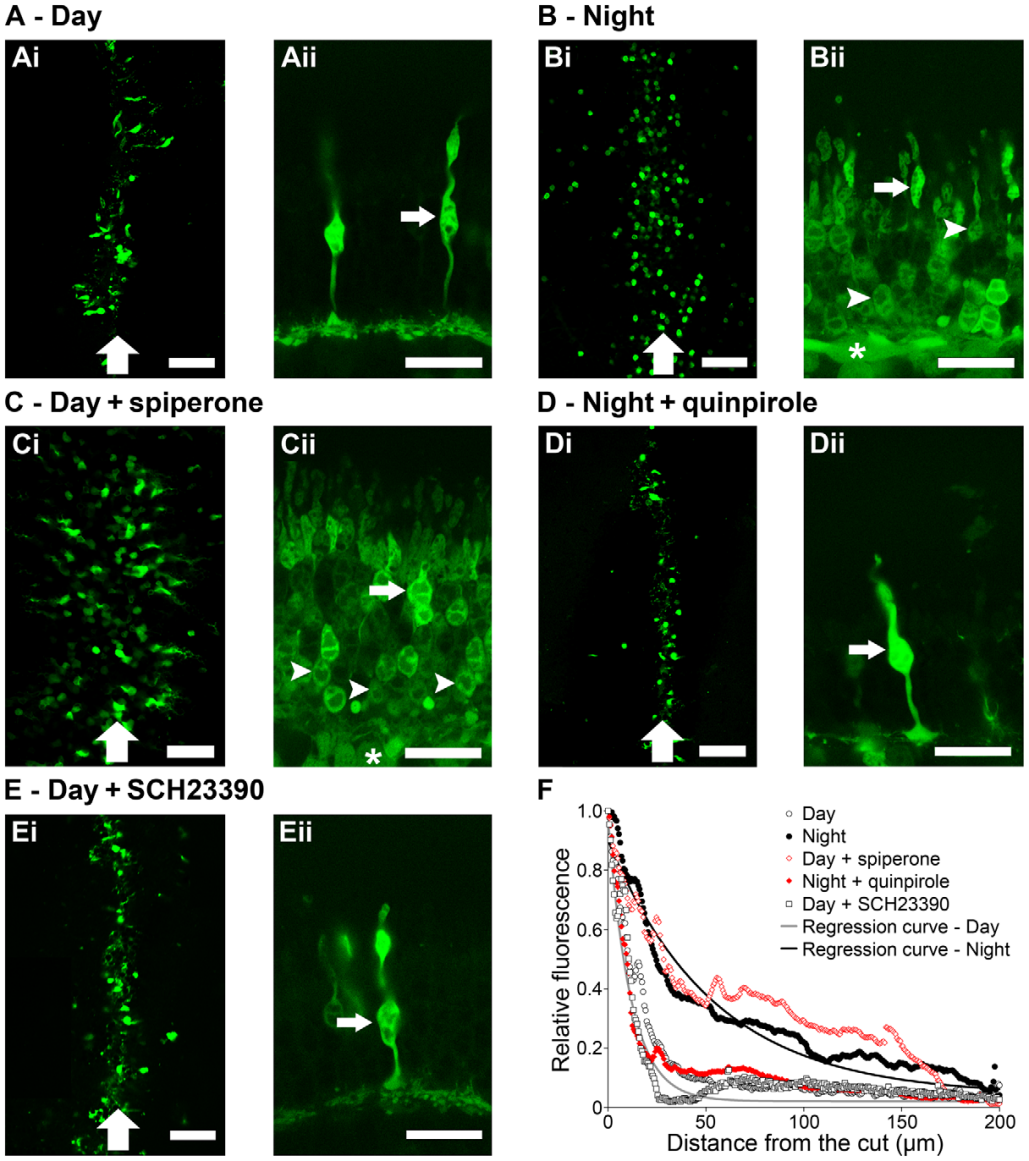
Tracer coupling between rabbit rod and cone photoreceptor cells varies with the time of day and D_2_ receptor activity. *A-E*, Typical examples of photoreceptor cell tracer coupling obtained under dark-adapted conditions during the day (*A*), night (*B*), and day in the presence of spiperone (10 µM) (*C*), night in the presence of quinpirole (1 µM) (*D*), and day in the presence of SCH23390 (10 µM) (*E*). Shown are confocal images of whole-mount rabbit retinas taken parallel to the retinal surface at the level of the photoreceptor inner segments near the cut (*Ai-Ei*) and detailed perpendicular views at higher magnification of the 3D reconstruction of the labeled photoreceptor cells (*Aii-Eii*). The micrographs in *Aii-Eii* show labeled photoreceptor cells in images that range along the horizontal axis from the cuts (leftmost edge of the micrographs) to 50 µm from the cuts (rightmost edge). In addition, at the bottom of the micrographs cone pedicles are visible in *Aii*, *Dii* and *Eii*, and horizontal cells/bipolar cells are indicated (asterisks) in *Bii* and *Cii* proximal to the photoreceptors. Large vertical arrows indicate the location of the cuts in *Ai-Ei*. Some cones (small arrows) and rods (arrowheads) are indicated in *Aii-Eii*. Rod cell bodies are located in the innermost half of the outer nuclear layer, whereas cone cell bodies are typically located in the outermost half of the outer nuclear layer [49]. Scale bar = 50 µm (*Ai-Ei*); 20 µm (*Aii-Eii*). *F*, Averaged normalized fluorescence in the photoreceptor cell layer as a function of the distance from the cut under dark-adapted conditions during the day (open circles; *n* = 6), night (filled circles; *n* = 4), and during the day in the presence of spiperone (open diamonds; *n* = 4) or SCH23390 (open squares; *n* = 2), and night in the presence of quinpirole (filled diamonds; *n* = 4). Curves generated from the non-linear analysis of the data during the day (grey curve) and night (black curve) are also shown. Data points represent averaged data from *n* experiments (1 retina/condition/experiment) ± SEM.

Because A-type horizontal cells make synaptic contact with cones and not with rods [1], [13], the increased rod input to A-type horizontal cells at night strongly suggests that rod-cone electrical coupling is increased at night in the rabbit retina, as has been observed in fish and mouse retinas [6], [19]. To determine whether rod-cone coupling in the rabbit is increased at night, we investigated whether the extent of tracer coupling between photoreceptors in the rabbit retina under dark-adapted conditions depends on the time of day using application of neurobiotin, which is gap junction permeable, but not membrane permeable [14], [16]. Neurobiotin diffusion, as revealed by the fluorescence of Alexa488, was restricted to the cells adjacent to the cut during the day (Fig. 6A and 6F, length constant (λ) = 11.61±0.13 µm) and during the night in the presence of quinpirole (1 µM; Fig. 6D and 6F, λ = 11.36±0.23 µm), but was observed at night (Fig. 6B and 6F, λ = 57.30±1.34 µm) and during the day in the presence of spiperone (10 µM; Fig. 6C and 6F, λ = 64.64±1.38 µm) (*P*<0.001; Tukey *post-hoc* test) in densely packed photoreceptor cells up to 80 µm from the cut and in less densely packed photoreceptor cells as far as 150 µm from the cut. Based on their morphology and the position of their somata in the outer nuclear layer, the labeled cells were identified as mostly cones during the day (Fig. 6Aii) and at night in the presence of quinpirole (Fig. 6Dii). In contrast, fluorescence was detected in both cones and rods during the night (Fig. 6Bii) and during the day in the presence of spiperone (Fig. 6Cii). The presence of the D_1_ antagonist SCH23390 (10 µM) did not affect the extent of photoreceptor tracer coupling (Fig. 6E and 6F, λ = 9.70±0.16 µm). The exponential decrease in fluorescence intensity as a function of distance from the cut in all cases examined (see Fig. 6F) indicates that the neurobiotin tracer entered the photoreceptors via the cut and not from other sites. These findings thus indicate that the retinal circadian clock uses D_2_, but not D_1_, receptor activation to control rod-cone tracer coupling, so that coupling is minimal during the day and extensive at night.

## Discussion

**Figure 7:**
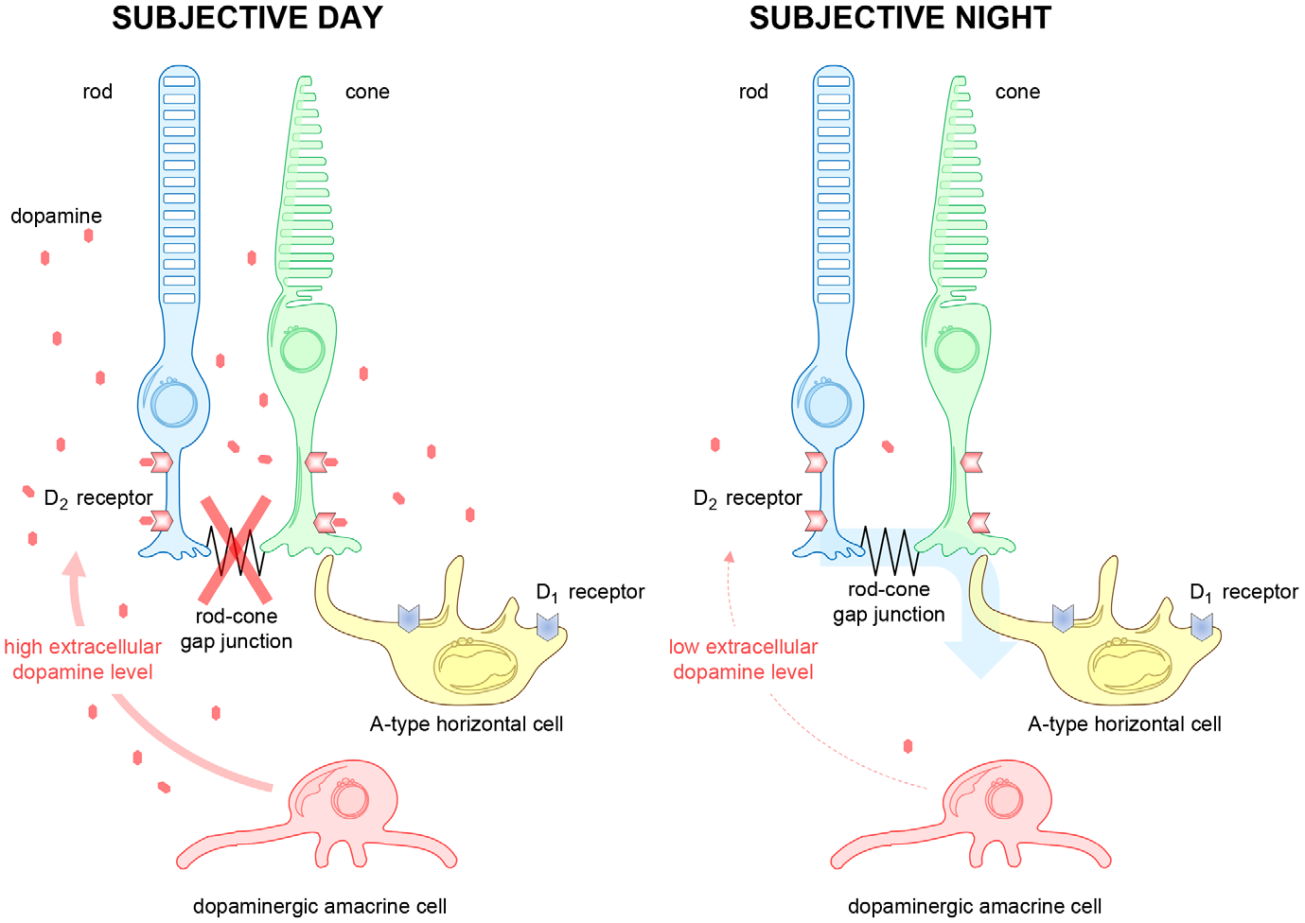
Schematic representation of circadian clock control of a neural pathway in the mammalian retina. The retinal clock increases dopamine release in the subjective day so that the dopamine D_2_ receptors on rods and cones are activated. This in turn greatly reduces the conductance of the gap junctions between rods and cones so that rod input to cones and cone-connected (e.g. axonless A-type) horizontal cells is minimal. In contrast, during the subjective night the retinal clock decreases endogenous D_2_ receptor activation, so that the conductance of rod-cone gap junctions is strong. As a result, under dark-adapted conditions dim light (scotopic range - see definition in Materials and Methods) stimuli evoke responses from rods, cones and cone-connected horizontal cells at night, but evoke responses only from rods in the day.

The findings in this study of A-type horizontal cell light responses and tracer coupling and rod-cone tracer coupling in the rabbit retina at different times of the day and night are the first to show that the circadian clock in the mammalian retina regulates the light responses of a specific retinal neuron (i.e. the A-type horizontal cell), and the first to identify a circadian-controlled rod pathway in the mammalian retina that functions at night, but not in the day. More specifically, our study resulted in three main findings. First, the circadian clock in the mammalian retina regulates the light responses of A-type horizontal cells by increasing rod input to these cells at night (Figs. 1–4, Table 1). Second, the clock controls the extent of rod-cone tracer coupling, so that tracer coupling is restricted to a few cells during the day and is extensive at night (Fig. 6). Third, the clock decreases both rod-cone tracer coupling and rod input to A-type horizontal cells in the day by increasing dopamine D_2_, but not D_1_, receptor activation in the outer retina (Figs. 1–4, Table 1). Together with previous work that showed that mammalian A-type (axonless) horizontal cells make synaptic contact with cones, but not with rods [1], [13], our findings strongly suggest that the rod-cone gap junction serves as a synaptic site in mammals through which the retinal clock controls signal flow from rods to cones and then to neurons postsynaptic to cones. As shown in Figure 7, the clock decreases D_2_ receptor activation at night, so that rod-cone coupling and rod input to A-type horizontal cells are robust, but the clock increases D_2_ receptor activation in the day, so that rod-cone coupling and rod input to A-type horizontal cells are minimal.

### Identification of a circadian clock-controlled rod pathway in the mammalian retina

We show here that rabbit A-type horizontal cells respond to very dim light stimuli (in the low scotopic range) at night, but not in the day. Although 1) rod-cone gap junctions have been observed in all vertebrate retinas, including mammalian (non-primate and primate) retinas, that contain both rods and cones [8], [20] and 2) dark-adapted mammalian horizontal cells have been reported to receive substantial rod input [21], [22], it has been accepted for more than twenty years, based on experimental observations and theoretical considerations [20], [23]–[27], that rod-cone coupling is minimal under very dark (low scotopic) conditions and that very dim light information from rods is not transmitted directly to cones. This weak rod-cone coupling has been thought to render the rod to cone to cone bipolar cell pathway less sensitive than the rod to rod bipolar cell to AII amacrine cell pathway. However, the difference in sensitivity between the two rod pathways may be much smaller than previously assumed. Specifically, using a 0.5-mV criterion, we found that the light response threshold of dark-adapted A-type horizontal cells is ∼1 Rh*.rod^−1^.s^−1^ during the day and ∼0.1 Rh*.rod^−1^.s^−1^ at night. Considering a rod integration time ∼200 ms, our data thus clearly support the view that very dim light signals in the low scotopic range (<0.1 Rh*.rod^−1^.s^−1^) may reliably reach cones from rods at night. Moreover, recent observations that monkey cones are able to detect brief light stimuli as dim as 0.5 Rh*.rod^−1^ due to their coupling to rods [28] and that a rod pathway in the rabbit retina, which is distinct from the rod to rod bipolar cell to AII amacrine cell pathway, is able to transmit very dim light stimuli (<0.2 Rh*.rod^−1^.s^−1^ [29]; <0.5 Rh*.rod^−1^.s^−1^, [30]) support this view. Thus, although isolated mammalian cones, which have been dissociated from the retina, do not respond to dim light (i.e. scotopic) stimuli, evidence strongly suggests that dark-adapted cones in the intact retina can detect very dim light stimuli and transmit these signals to second-order neurons at night due to the strong rod-cone coupling. According to this view, previous studies, which did not report low scotopic rod signals in the cone pathways (i.e. horizontal cells, ganglion cells), were likely not performed at night under dark-adapted conditions.

In addition to increasing the direct transmission of rod signals into cones, the increase in rod-cone coupling at night may also enhance the detection and transmission of weak signals in rod pathways in response to very dim large objects. Because intrinsic noise in a photoreceptor cell is independent of the noise in its neighbors, but responses of neighboring photoreceptor cells to dim large objects are correlated, photoreceptor cell coupling at night will reduce photoreceptor cell noise more than it decreases their light responses to large dim objects [31]. Thus, the circadian-controlled increase in photoreceptor cell coupling at night augments the signal to noise ratio of rod responses to very dim large objects before the signal and noise are distorted by the highly nonlinear rod to rod bipolar cell synapse [27], resulting in a more reliable signal through the rod pathways. The circadian-induced increase in photoreceptor cell coupling at night therefore enhances nighttime vision, which is characterized by high sensitivity and low acuity, and the decrease in photoreceptor cell coupling in the day augments daytime vision, which is characterized by low sensitivity and high acuity [32].

The findings reported here on rabbit retina, together with recent studies on fish and mouse retinas [6], [7], [19], [33]–[35] strongly suggest that circadian clock regulation of rod-cone coupling and of rod input to cones and cone-connected second-order cells is conserved in most, if not all, mammalian and non-mammalian vertebrates that have both rod and cone photoreceptors (duplex retinas) because in both mammalian and non-mammalian retinas 1) there is a circadian clock that increases dopamine release in the day [4], [5], 2) rod-cone gap junctions have been observed [8], [20]; 3) rods and cones express D_2_ receptors and horizontal cells express D_1_ receptors [36]; 4) rod-cone coupling is greater at night than in the day and is regulated by D_2_, but not D_1_, receptors (Fig. 6) [6], but coupling between cone-connected horizontal cells is regulated by D_1_ receptors and does not exhibit a day/night difference (Fig. 5) [34], and 5) cone-connected horizontal cells, which have chemical synaptic contact with cones, but not rods [9], [10], [13], and fish cones respond to light stimuli in the low scotopic range at night, but not in the day, due to D_2_ receptor activation in the day (Figs. 1–4) [6], [7], [33], [35]. In addition, the effects of dopamine on rod-cone coupling are likely mediated in part by cAMP and cAMP-dependent phosphorylation of connexin 35/36 [7], [19]. Although it is possible that the retinal clock increases the conductance of cone-cone and/or rod-rod gap junctions at night, in addition to increasing rod-cone coupling, the increase in the conductance of rod-cone gap junctions at night would effectively increase electrical and cellular communication between cones and cones and between rods and rods, as well as between rods and cones, at night. Although the day-night differences in rod input to horizontal cells and in rod-cone coupling have been observed in both rabbits and fish, under dark-adapted conditions rabbit A-type horizontal cell light responses are larger in amplitude at all intensities at night than in the day (Figs. 1, 2), but fish H1 (cone-connected) horizontal cell light responses to bright lights are smaller in amplitude at night than in the day [7], [33], [35], suggesting a species difference in circadian regulation of cone to horizontal cell synaptic transmission.

As has been previously suggested [6], [34], the findings that rod-cone coupling and rod input to horizontal cells, but not coupling between horizontal cells (see Fig. 5), exhibit a day/night difference that is dependent on D_2_ receptor activation can be explained by the difference in the affinity of D_1_ and D_2_ receptors for endogenous dopamine in the retina [7], [36] and elsewhere in the brain [37]. Specifically, the retinal clock increases extracellular dopamine levels in the outer retina sufficiently to activate the high affinity D_2_ receptors on rods and cones, but not enough to activate the low affinity D_1_ receptors on horizontal cells.

What role do melanopsin ganglion cells play, if any, in the day-night differences in rod input to A-type horizontal cells and rod-cone tracer coupling that we have observed under dark-adapted conditions and reported in this study? Because melanopsin ganglion cells regulate day-night differences in the amplitude and speed of the mouse electroretinogram under light-adapted conditions [38], it is possible that diurnal differences in cone pathway function under light-adapted conditions are melanopsin-dependent. In addition, melanopsin ganglion cells may play a role in the control of the light-evoked release of dopamine [39], [ but see 40]. However, it remains somewhat speculative as to whether melanopsin ganglion cells are involved in the circadian clock control of rod pathway function (i.e. rod input to A-type horizontal cells and rod-cone tracer coupling) under dark-adapted conditions.

Circadian clock control of electrical coupling may have significant functional consequences in the retina and elsewhere in the brain. Specifically, given the abundance of electrical synapses in other brain areas [41], such as the cerebral cortex, thalamus and hippocampus, and the widespread control of brain activity by circadian clocks [42], our results suggest that circadian clock control of the conductance of electrical synapses [43] may be a common and important means by which neural signaling is modulated in the brain.

In summary, rod input reaches rabbit A-type horizontal cells at night via rod-cone gap junctions, which are opened by the circadian clock in the mammalian retina. In the day, the clock-induced increase in D_2_ receptor activation decreases rod-cone coupling, so that rod input to the horizontal cells is minimal. These results demonstrate that the rod-cone gap junction serves as a synaptic site in mammals through which the retinal clock controls signal flow from rods to cones and to neurons postsynaptic to cones. The findings thus identify a circadian clock-controlled rod pathway in the mammalian retina that functions at night, but not in the day, and suggest that the retinal clock plays a fundamental role in the twice-daily transition at dawn and dusk between day and night vision in mammals.

## Materials and Methods

### Animal care and use/Tissue preparation

All experimental procedures were performed in accordance with the guidelines of the National Institutes of Health on the care and use of experimental animals. All experimental procedures involving the care and use of rabbits in this study were reviewed and approved by the Ohio State University Institutional Animal Care and Use Committee (PHS Animal Welfare Assurance No. A3261-01). The *in vitro* rabbit retina was used in this circadian study as a model mammalian retina for two reasons. First, although the neural retinas of most mammalian species, including mouse, are thick and vascular, the rabbit neural retina is thin and avascular with capillaries on either side that provide needed nutrients by diffusion [44]. As a result, the superfused *in vitro* rabbit retina can be more easily maintained in a viable healthy state for many hours, as was needed for our circadian study. Second, although both rabbit and mouse retinas have axon-bearing (B-type) horizontal cells, which have dendrites that are postsynaptic to cones and axon terminals that are postsynaptic to rods [1], only the rabbit retina also contains axonless (A-type) horizontal cells, which have dendrites that make synaptic contact exclusively with cones [1], [13]. We have investigated the light responses of A-type, rather than B-type, horizontal cells in our circadian study, because 1) it is easier to record the light responses of A-type horizontal cells under conditions of constant darkness in the day and night due to the larger size of their somata compared to that of B-type cells [1], [11]–[14], [16] and 2) the presence of a rod component at night in the light responses of A-type horizontal cells, which make synaptic contact exclusively with cones [1], [13], can be unambiguously interpreted as due to the flow of rod signals into cones through open rod-cone gap junctions, and not due to a direct rod to A-type horizontal cell pathway.

Following deep general (urethane, loading dose: 2.0 g/kg, I.P.) and local intraorbital (2% Xylocaine) anaesthesia, experiments were performed on the superfused, Dutch-belted (pigmented) adult rabbit eyecup preparation, as described previously [45]. The neural retina attached to the epithelium-sclera was used for electrical recording experiments and the isolated neural retina was used for cut-loading experiments (see below).

Before experiments, the rabbits were maintained for at least 2 weeks on a 12 h light/12 h dark cycle with lights-on at 4.00 a.m. Rabbits were dark-adapted for at least 1 h before all experiments. Rabbits were kept in darkness for 24–48 h before circadian experiments. Surgery was performed under infrared illumination. Eyecups were superfused for 60 min in the dark before the start of electrical recording. The phrases “subjective day” and “subjective night” refer to the day and night of the imposed light/dark cycle, respectively, when animals or isolated retinas in circadian experiments were maintained in constant darkness.

### Lighting conditions

A 100 W tungsten-halogen lamp provided light for a single beam optical bench that provided full-field light stimulation. The unattenuated intensity (*I*_o_) at the retinal surface was 2.0 mW.cm^−2^.s^−1^. Intensity values indicated in the text are relative to *I*_o_. During all circadian and dark adaptation electrical recording/tracer injection and cut-loading experiments in the day and night, the background illumination was <−11 log *I*_o_ (i.e. >4.5 log units lower than daytime A-type horizontal cell threshold). Calibrated neutral density filters and narrow-band interference filters were used to control light intensity and stimulus wavelength, respectively. The term “photopic” refers to the range of bright ambient light, which typically occurs during a sunny day, to which cones, but not rods, can respond. In contrast, the term “scotopic” refers to the range of very dim ambient light, which typically occurs during a moonless night, to which rods, but not cones, which have been separated from the retina, can respond. Lastly, the term “mesopic” refers to the range of ambient light between the scotopic and photopic ranges, which typically occurs at dawn and dusk, to which both rods and cones can respond.

Spectral sensitivity data were corrected for equal energy and a 0.5-mV response criterion was used to minimize light adaptation of dark-adapted retinas. The maximum, unattenuated photon density of the stimulus at 500 nm (*I*_o–500_) was 5.01.10^13^ photons.cm^−2^.sec^−1^. Photon density was converted to mean isomerization rate per rod (Rh*.rod^−1^.s^−1^) from an average density of rod photoreceptors (d_rod_) in the rabbit visual streak ∼250,000.mm^−2^ [46], a quantum efficiency of absorption (Q_eff_Ab) of 20% [29] and a quantum efficiency of isomerization (Q_eff_Iso) of 67% [47], and according to the expression:
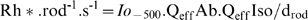



Thus, the unattenuated photon density at 500 nm was equivalent to ∼2.73.10^5^ Rh*.rod^−1^.s^−1^. The peak sensitivity (*k*) was converted to mean isomerization rate per rod according to the expression:
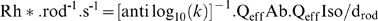



### Electrical recordings of rabbit horizontal cells

Standard intracellular recording procedures were employed. Pipettes were filled with 4% neurobiotin (Vector Laboratories, Burlingame, CA) in 0.1 M TRIS and backfilled with 4 M KCl. All impalements were made in or near (∼±2 mm) the visual streak without the aid of any light.

### Definitions of response measures

We defined the response latency as the time between light stimulus onset and the beginning of the hyperpolarizing response, defined as a downward deflection of the membrane potential equal to at least two times the amplitude of the noise. The time to peak was defined as the time between the beginning of the light response and its maximum amplitude (i.e. the most negative value of the membrane potential); the response amplitude was the difference between the resting membrane potential and the membrane potential at the peak response; and the duration of the response was the duration of the hyperpolarization. Indeed, the appearance of a depolarizing component at the end of the light response was not consistent from cell to cell and was not analyzed further. The end of the response was thus set as the time the membrane potential equaled the initial resting potential for the first time following the light-evoked hyperpolarization.

### Morphological identification of A-type horizontal cells and tracer coupling

The morphology of the recorded cells was revealed by injection and cytochemical visualization of the biotinylated tracer Neurobiotin, as described elsewhere [6], [14], [16], [17].

### Cut-loading experiments

Cut-loading was performed as described elsewhere [6], [19], except that the razor blades were dipped in neurobiotin (0.5%) right before the retinas were cut. Specifically, several perpendicular radial cuts of rabbit neural retinas were made with a razor blade immediately after isolation of the retinas under dark-adapted conditions. The retinas were then incubated for 15 min in the bicarbonate-buffered saline solution. Following cell loading and diffusion, the retinas were then washed in saline and fixed in 4% paraformaldehyde in 0.1 M phosphate buffer (pH 7.3) for 1 hr. Neurobiotin was visualized with strepavidin-conjugated-Alexa488 (Molecular Probes, Eugene, OR). In some experiments, the retinas were isolated and incubated in saline with spiperone (10 µM), quinpirole (1 µM), or SCH23390 (10 µM) for 30 min before the cuts were made. Drug was present during the subsequent steps as well as until fixation.

### Statistical analysis

Statistical analyses were performed using Origin 7.0 SR4 (OriginLab Corporation, Northampton, MA).

Normalized light response peak amplitude data were fit to a Hill-type equation in the form:
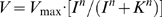
where *V* is the response amplitude, *V*_max_ is the maximum response amplitude, *I* is the stimulus intensity, *K* is the stimulus intensity needed to generate a response with half-maximal amplitude, and n is the Hill coefficient. Nonlinear least-squares regression analysis was performed with n and *K* as free parameters.

Statistical analysis of A-type horizontal cell spectral sensitivity was done as described previously [6], using nonlinear least-squares regression of our experimental data with the published template for a mammalian vitamin A_1_-based visual pigment, with the peak sensitivity (*k*) and the wavelength at the peak sensitivity (*λ*_max_, nm) as free parameters [48]. For relative spectral sensitivity data analysis, data were normalized to the maximum sensitivity and *k* was set to 0.

For the cut-loading experiments, cells were imaged and photographed with a Zeiss 510 META laser scanning confocal microscope (Carl Zeiss, Inc., Thornwood, NY). Serial reconstructions of rods and cones were made from z-stacks of confocal images with LSM-5 Image Browser 3,2,0,115 (Carl Zeiss). Rods and cones could be clearly distinguished in z-stacks of whole-mount sections based on their morphology and the position of their somata in the outer nuclear layer. Fluorescence intensity of Alexa488-labeled Neurobiotin was measured from low-magnification images of whole-mount retinas using the NIH ImageJ software. No distinction was made between the photoreceptor types and the data were normalized to the maximum fluorescence intensity and fit to a first-order exponential decay function in the form:
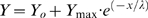
where *Y* is the relative fluorescence intensity, *Y_0_* is the background fluorescence, *Y_max_* is the maximal relative fluorescence, *λ* is the length constant, and *x* the distance from the cut. Nonlinear least-squares regression analysis was performed with *Y_0_*, *Y_max_*, and *λ* as free parameters.

Results from the least-squares nonlinear regression analysis are given ± standard deviation (s.d.). To compare 2 groups of data, statistical analysis was performed using the unpaired-Student's *t*-test. To compare more than 2 groups, statistical analysis was performed using one-way or two-way analysis of variance (ANOVA). We used Tukey's multiple comparison post test.
